# Involvement of Phosphatidylinositol 3-kinase in the regulation of proline catabolism in *Arabidopsis thaliana*

**DOI:** 10.3389/fpls.2014.00772

**Published:** 2015-01-12

**Authors:** Anne-Sophie Leprince, Nelly Magalhaes, Delphine De Vos, Marianne Bordenave, Emilie Crilat, Gilles Clément, Christian Meyer, Teun Munnik, Arnould Savouré

**Affiliations:** ^1^Sorbonne Universités, Universite Pierre et Marie Curie Univ Paris 06, Adaptation de Plantes aux Contraintes Environnementales, URF5Paris, France; ^2^INRA-AgroParisTech, Institut Jean-Pierre Bourgin, UMR 1318, ERL CNRS 3559, Saclay Plant SciencesVersailles, France; ^3^Section Plant Physiology, Swammerdam Institute for Life Sciences, University of AmsterdamAmsterdam, Netherlands

**Keywords:** *Arabidopsis thaliana*, lipid signaling, Phosphatidylinositol 3-kinase (PI3K), proline, proline dehydrogenase 1 (ProDH1), salt stress

## Abstract

Plant adaptation to abiotic stresses such as drought and salinity involves complex regulatory processes. Deciphering the signaling components that are involved in stress signal transduction and cellular responses is of importance to understand how plants cope with salt stress. Accumulation of osmolytes such as proline is considered to participate in the osmotic adjustment of plant cells to salinity. Proline accumulation results from a tight regulation between its biosynthesis and catabolism. Lipid signal components such as phospholipases C and D have previously been shown to be involved in the regulation of proline metabolism in *Arabidopsis thaliana*. In this study, we demonstrate that proline metabolism is also regulated by class-III Phosphatidylinositol 3-kinase (PI3K), VPS34, which catalyses the formation of phosphatidylinositol 3-phosphate (PI3P) from phosphatidylinositol. Using pharmacological and biochemical approaches, we show that the PI3K inhibitor, LY294002, affects PI3P levels *in vivo* and that it triggers a decrease in proline accumulation in response to salt treatment of *A. thaliana* seedlings. The lower proline accumulation is correlated with a lower transcript level of *Pyrroline-5-carboxylate synthetase 1* (*P5CS1*) biosynthetic enzyme and higher transcript and protein levels of *Proline dehydrogenase 1* (*ProDH1*), a key-enzyme in proline catabolism. We also found that the *ProDH1* expression is induced in a *pi3k*-hemizygous mutant, further demonstrating that PI3K is involved in the regulation of proline catabolism through transcriptional regulation of *ProDH1*. A broader metabolomic analysis indicates that LY294002 also reduced other metabolites, such as hydrophobic and aromatic amino acids and sugars like raffinose.

## Introduction

As sessile organisms, plants need to cope with adverse environmental stresses. Abiotic constraints such as drought and salinity have a major impact on plant development and crop productivity (Zhu, 2002). A common feature of drought and salt stress is the lower availability of water, due to decrease of soil water potential. In addition, salt generates an ionic stress due to the presence of Na^+^ and Cl^−^. Perception of drought and salt constraints triggers complex signaling networks, which then induce the adaptive response of plants. Among these networks, various molecular components are involved, including phytohormones, protein kinases and phosphatases, and second messengers like Ca^2+^, ROS, and lipid signaling elements (Munnik and Vermeer, 2010; Huang et al., 2012; Deinlein et al., 2014; Golldack et al., 2014).

Phospholipids are important structural components of cellular membranes but can also play an essential role in the adaptation of plants to abiotic stress (Munnik and Testerink, 2009; Xue et al., 2009; Munnik and Vermeer, 2010; McLoughlin and Testerink, 2013). They are modified by enzymes such as phospholipase C (PLC) and D (PLD), and by lipid-kinases, such as diacylglycerol kinase (DGK), PA kinase and various phosphoinositide kinases (Meijer and Munnik, 2003). These modifications produce important second messengers that regulate various plant responses.

Phosphatidylinositol 3-kinase (PI3K) phosphorylates the D-3 position of the inositol ring of phosphoinositides. In mammals, three distinct PI3K classes (I-III) can be distinghuised, differing in gene structure, enzyme regulation, and substrate preference. Class III PI3Ks are homologous to the yeast VPS34, which uses PI as a sole substrate to produce PI3P (Backer, 2008). VPS34 promotes membrane fusion and vesicle trafficking by recruiting PI3P-binding proteins to membranes. VPS34 is associated with different proteins, forming distinct protein complexes, including the regulation of mTORC1 (Target of rapamycin complex 1) that monitors the nutritional status of the cell (Backer, 2008; Ktistakis et al., 2012; Robaglia et al., 2012).

Higher plants only contain VPS34-like PI3Ks (Lee et al., 2010). In Arabidopsis, PI3K activity is encoded by a single gene (At1g60490), which is important for pollen development (Welters et al., 1994; Lee et al., 2010). Pollen grains harboring a *pi3k* KO allele display abnormal germination (Lee et al., 2008b; Gao and Zhang, 2012), preventing the acquirement of homozygous *pi3k* KO mutants. PI3P has been detected in vacuolar membranes and in late endosomal and pre-vacuolar compartments, indicating that, like in mammals and yeast, plant PI3K is involved in vesicle trafficking and membrane biogenesis (Voigt et al., 2005; Vermeer et al., 2006; Lee et al., 2010; Simon et al., 2014). PI3P is considered as a second messenger that recruits PI3P-binding proteins to membranes (Meijer and Munnik, 2003; Van Leeuwen et al., 2004; Wywial and Singh, 2010). Numerous data indicate that PI3K and its product PI3P are involved in plant responses to drought and salt stress. PI3P participates in stomata closure in response to abscisic acid (ABA) and ROS production in guard cells (Jung et al., 2002; Park et al., 2003; Choi et al., 2008). Also NADPH oxidase endocytosis leading to ROS production upon ionic stress is triggered by PI3K via PI3P (Leshem et al., 2007).

In plants, PI3P can be further phosphorylated by PI3P-5 kinase (FAB) to produce PI(3,5)P_2_ (Meijer et al., 1999; Munnik and Nielsen, 2011; Gao and Zhang, 2012).

Wortmannin (Acaro and Wymann, 1993) and LY294002 (Vlahos et al., 1994) are pharmacological PI3K inhibitors that have frequently been used to decipher the function of PI3Ks and their products in various mammalian and yeast systems. LY294002 is derived from the flavonoid quercetin and competes with ATP and binds to a Lys residue in the ATP-binding pocket of PI3Ks (Walker et al., 2000). As such, LY294002 has been described to inhibit PI3K-related kinases such as TOR (Target of rapamycin) and DNA-PK (DNA-dependent protein kinase; Brunn et al., 1996), but also other protein kinases, such as Casein Kinase 2 (Gharbi et al., 2007). Nevertheless LY294002 is considered to be a more selective PI3K inhibitor (Walker et al., 2000; Jung et al., 2002; Templeton and Moorhead, 2005).

In plants, Wortmanin and LY294002 have also been used to show the involvement PI3K and PI3P (Jung et al., 2002; Park et al., 2003; Jallais et al., 2006; Vermeer et al., 2006; Leshem et al., 2007; Choi et al., 2008; Lee et al., 2008a; Takáč et al., 2012, 2013). LY294002 was shown to inhibit stomatal closing induced by ABA, polar tip-growth of root hairs, and chloroplast accumulation in response to blue light (Jung et al., 2002; Lee et al., 2008a; Aggarwal et al., 2013). At the subcellular level, LY294002 blocks endocytosis and vacuolar trafficking and inhibits auxin-mediated ROS generation (Etxeberria et al., 2005; Joo et al., 2005). Despite LY294002's frequent use, only few reports have showed an effect on PI3K activity *in vitro* (Jung et al., 2002; Joo et al., 2005), yet none *in vivo*.

In response to water stress, plants accumulate organic osmolytes such as amino acids, sugars and polyamines, which play key roles in decreasing the cellular osmotic potential but also in preventing the aggregation and/or precipitation of macromolecules following the decrease of water availability (Slama et al., 2015). Among these organic osmolytes, free proline is well known to rapidly increase upon water constraints (Szabados and Savouré, 2010; Liang et al., 2013). Proline levels represent a delicate balance between biosynthesis and catabolism. Proline is synthetized from glutamate by a two-step reaction (Szabados and Savouré, 2010; Liang et al., 2013). First, glutamate is reduced to glutamyl-5-semialdehyde (GSA) by the bifunctional enzyme pyrroline-5-carboxylate synthetase (P5CS). GSA is then spontaneously converted to P5C, which is then reduced to proline by P5C reductase (P5CR). The rate-limiting enzyme of the biosynthetic pathway is P5CS, which is encoded by two genes *P5CS1* and *P5CS2* in *A. thaliana*. These two isoforms play distinct roles during development and stress responses (Székely et al., 2008). Under normal growth conditions, proline biosynthesis occurs in the cytosol and is mainly under the control of P5CS2. *P5CS2* has been shown to be expressed in dividing cells, in meristematic and reproductive tissues (Strizhov et al., 1997; Székely et al., 2008; Mattioli et al., 2009). Upon salt stress and drought, proline accumulation is dependent of *P5CS1* expression (Savouré et al., 1995; Yoshiba et al., 1995). P5CS1 has been shown to be localized in chloroplasts upon water stress by Székely et al. (2008).

Upon relief from stress, proline is rapidly oxidized in mitochondria by a two-step reaction. First, proline is oxidized by proline dehydrogenase (ProDH) to form P5C, which is then oxidized to glutamate by P5C dehydrogenase (P5CDH). ProDH is the rate-limiting enzyme for proline catabolism and is encoded in Arabidopsis by two genes, *ProDH1* (also named *ERD5*) and *ProDH2* (Servet et al., 2012). ProDH1 is considered as the main isoform, *ProDH2* being weakly expressed (Kiyosue et al., 1996; Funck et al., 2010). Under either salt or drought stress, *ProDH1* expression is repressed allowing proline accumulation (Funck et al., 2010). On the opposite, when stress is relieved, *ProDH1* expression is triggered leading to proline degradation in mitochondria (Kiyosue et al., 1996; Verbruggen et al., 1996).

Proline accumulation in response to water stress is not only important for osmotic adjustment, but also as scavenger for reactive oxygen species (ROS) and molecular chaperone to stabilize proteins, antioxidant enzymes and membrane structures (Szabados and Savouré, 2010; Liang et al., 2013). Proline is also considered as a source of energy, which may be important upon stress recovery (Szabados and Savouré, 2010; Liang et al., 2013; Kavi Kishor and Sreenivasulu, 2014).

As proline accumulation and degradation result from a tight regulation of its metabolism, deciphering the signaling networks involved is of prime importance. Activation of proline biosynthesis is linked to both ABA mediated-signal transduction (Strizhov et al., 1997; Abrahám et al., 2003) and ABA-independent signaling (Savouré et al., 1997; Sharma and Verslues, 2010). *P5CS1* expression was also shown to be positively regulated by ROS, acting as intermediate in ABA-mediated proline accumulation, while ProDH activity was repressed (Yang et al., 2009).

In Arabidopsis, lipid signaling components are involved in the regulation of *P5CS1* expression. Under normal growth condition, PLD negatively regulates *P5CS1* expression, preventing proline accumulation (Thiery et al., 2004). Upon ionic but not osmotic stress, PLC triggers *P5CS1* expression leading to proline accumulation (Parre et al., 2007). *P5CS1* up-regulation by PLC involves Ca^2+^ as a second messenger, which acts as a molecular switch to trigger downstream signaling events (Parre et al., 2007). Expression of both *ProDH* genes is regulated by bZIP transcription factors (Weltmeier et al., 2006; Hanson et al., 2008). After dark treatment or in response to hypoosmolarity stress, *ProDH1* expression is induced by the heterodimer bZIP53/bZIP10 which recognizes the ACTCAT regulating sequence in *ProDH1* promoter (Satoh et al., 2004; Weltmeier et al., 2006; Dietrich et al., 2011).

Here, the role of PI3K in the regulation of proline accumulation was investigated. Using a pharmacological and biochemical approach, we show that the decrease of PI3P by LY294002 treatment correlated with lower proline accumulation upon salt stress in Arabidopsis seedlings. The decrease of proline content was associated with both the reduction of *P5CS1* transcript and protein levels and the induction of *ProDH1* transcript and protein levels. In a reverse genetic approach, using a hemizygous *pi3k* mutant, a similar pattern of *ProDH1* expression was found as WT seedlings treated with LY294002. During normal growth condition, a strong expression of *ProDH1* is detected in WT seedlings treated with LY294002 as well as in *pi3k* hemizygous mutant. These data suggest that a signaling pathway involving PI3P participates to the regulation of proline metabolism in normal growth condition and in response to salt stress through the repression of proline catabolism. A detailed metabolite profiling analysis was conducted to search for other compounds regulated by PI3P. Interestingly, raffinose exhibited a similar pattern of accumulation as proline in the presence of LY294002. In addition, hydrophobic- and aromatic amino acid contents strongly increased in presence of LY294002.

## Materials and methods

### Plant material

Arabidopsis (*Arabidopsis thaliana*) Heynh, ecotype Columbia Col-0 as wild-type (WT) and *pi3k* hemizygous mutant from GABI library (GK_418H02-018138) were used. In the hemizygous *pi3k* mutant (*PI3K/pi3k*), T-DNA insertion is located in the fifth exon of one allele of the gene (Lee et al., 2008b). WT seeds were sown on 0.5× Murashige and Skoog (MS) solid medium (0.8% agar) in 14-cm-diameter Petri dishes as described previously (Parre et al., 2007). *pi3k* mutant seeds were sown on 0.5× MS solid medium supplemented with 19.2 μM sulfadiazine (dissolved in DMSO) in order to select hemizygous plants versus WT homozygous plants. After 16 h at 4°C to raise dormancy, seeds were germinated and grown under continuous light with an intensity of 90 μmole photons m^−2^ s^−1^ for 12 days at 22°C.

### Stress and pharmacological treatments

Twelve-days-old seedlings were removed from 0.5× MS agar plates and put onto liquid 0.5× MS medium (control) supplemented with either 200 mM NaCl or 400 mM mannitol. After different incubation times, seedlings were collected and immediately frozen in liquid nitrogen and stored at −80°C prior analysis.

For pharmacological treatments, seedlings were pre-treated for 1 h in 0.5× MS liquid medium with various concentrations of LY294002 dissolved in DMSO or with the same amount of DMSO as a control. Seedlings were thereafter transferred for 3 h or 24 h onto 0.5× MS liquid medium alone (control), or supplemented with either 200 mM NaCl or 400 mM mannitol and with the same amount of DMSO or LY294002 as for the pre-treatment.

### Proline determination

Free proline contents were measured using L-proline as a standard according to Bates et al. (1973).

### Phospholipids analysis

For practical reasons this experiment was performed on 3- to 5-days-old seedlings into a 2 mL Eppendorf tube containing 200 μL of 2.5 mM MES/KOH buffer (pH 5.7) and 1 mM KCl. In order to label phospholipids, 10 μCi of ^32^P-inorganic phosphate were added in each tube and incubated overnight (Munnik and Zarza, 2013). Either 100 μM LY294002 or the same amount of DMSO for the control were then added for 1 h of pre-incubation. Then a volume of 2.5 mM MES/KOH buffer (pH 5.7), 1 mM KCl buffer with 400 mM NaCl was added into the tube to reach a final concentration of 200 mM NaCl. For control condition, an equivalent volume of MES/KCl buffer was added. Treatments were stopped just after the addition of NaCl (0 h), or after 30 min or 3 h, by adding perchloric acid (5% w/v, final concentration), and after 10 min shaking the total solvent was removed. To extract lipids from the seedlings, 400 μl CHCl_3_ /MeOH/HCl (50/100/1, v/v/v) was added and the mix was vortexed for 10 min. To induce the separation of two phases, 400 μL CHCl_3_ and 200 μL 0.9% (w/v) NaCl were added, vortexed 10 s and then centrifuged for 1 min at 10,000 g. The organic lower phase was transferred to a new tube containing 400 μL CHCl_3_/MeOH/1M HCl (3/48/47, v/v/v). After shaking and centrifugation, the upper phase was removed, and 20 μL isopropanol was added to the purified organic phase, which was then dried down in a vacuum centrifuge at 50°C. The residue was dissolved in 100 μL CHCl_3_.

Phospholipids were separated as previously described (Munnik et al., 1994, 1995; Munnik and Zarza, 2013) by thin-layer chromatography (TLC) using heat-activated silica gel plates impregnated with a solution of 1% K-oxalate, 2 mM EDTA in MeOH/H_2_0 (2/3, v/v) and chromatographed with an alkaline solvent CHCl_3_/MeOH/NH_4_OH/H_2_O (90/70/4/16, v/v/v/v). Radiolabeled phospholipids were visualized and quantified using a phosphoImager.

In order to separate PI3P and PI4P, spots corresponding to the PIP pool was scraped off from the TLC plate and deacylated with 800 μL of mono-methylamine reagent (25% mono-methylamine/MeOH/n-ButOH (42.8/45.7/11.5, v/v/v) at 53°C for 30 min as described in (Munnik et al., 1994, 1996; Munnik, 2013). Samples were centrifuged at 10,000 g for 2 min and the supernatant was collected and dried under a N_2_ stream for 30 min and then by rotary evaporation. To remove the fatty-acyl groups, samples were dissolved in 500 μL H_2_O and extracted twice with 600 μL *n*-ButOH/petroleum ether (40–60°)/ethyl formate (20/40/1, v/v/v). The aqueous lower phase that contains glycerophosphoinositides (GroPInsP) was dried by rotary evaporation, dissolved in 500 μL H_2_O and filtered (0.22 μm).

GroPIns3P and GroPIns4P were separated by anion-exchange HPLC using a Partisil 10-SAX column and a discontinuous gradient of 1.25 M NaH_2_PO_4_ (pH 3.7) at a flow rate of 1 ml.min^−1^ (Munnik, 2013). Fractions were collected every 30 s and measured for radioactivity by liquid-scintillation counting.

### Northern blot analysis

Total RNAs were isolated from seedlings ground in liquid nitrogen using the guanidinium thiocyanate-CsCl purification method (Sambrook et al., 1989). RNAs were separated by electrophoresis in a 1.2% agarose-formaldehyde gel. After transfer to nylon membrane, RNAs were fixed by UV cross-linking. Membranes were hybridized at 65°C with either specific 3′UTR region of *AtP5CS1* or with full length of *AtProDH1* according to Church and Gilbert (1984). The fragments were labeled with ^32^P-dCTP using Ready-To-GoTM DNA labeling beads. Before hybridization, membranes were stained with methylene blue as a control for RNA loading and transfer. The hybridization signals were quantified using a PhosphorImager (Amersham Biosciences, USA).

### Quantitative RT-qPCR analysis

Total RNAs were extracted following the protocol of the RNeasy Plant Mini Kit (Qiagen) from around 100 mg of powder obtained after grinding a pool of seedlings. After treatment with the RNase-free DNase (Fermentas), 1.5 μg of total RNA were reverse-transcribed by Revertaid™ Reverse Transcriptase (Fermentas) using 1 μM oligodT following the manufacturer instructions. The resulting first-strand cDNA was 20-fold diluted and used as the template for real-time quantitative PCR (RT-qPCR) amplification performed on a MasterCycler®ep realplex thermocycler (Eppendorf) with Maxima® SYBR Green/ROX qPCR Master Mix (Thermo Scientific) following the manufacturer protocol. Each reaction was performed with 5 μL diluted cDNA sample in a total reaction volume of 15 μL. The relative expression of *P5CS1* (At2g39800), *ProDH1* (At3g30775) and *PI3K* (At1g60490) genes were determined using specific primers (Supplementary Table 1). Expression levels of the different genes were standardized to *APT1* (At1g27450) used as a standard reference. The applied RT-qPCR program was 2 min at 95°C, 40 cycles with 15 s at 95°C, 30 s at 57°C and 30 s at 72°C followed by 15 s at 95°C, 15 s at 55°C, a gradual temperature rise of 20 min to 55°C at 95°C associated with a streaming of the plate, followed by 15 s at 95°C. The expression level of each gene was calculated using the following equation: 2^(CtAPT1−Ctgene)^ × 100.

### Gel electrophoresis, electro-blotting and immunological detection

Proteins were extracted as described in Martínez-García et al. (1999) separated by SDS-PAGE (Laemmli, 1970) and transferred electrophoretically to a nitrocellulose membrane in a solution of 48 mM Tris, 39 mM glycine, 0.04% (w/v) SDS and 20% (v/v) ethanol at 50 mA for 1 h. For immunodetection, the nitrocellulose membrane was incubated in TBS with 0.05% (v/v) Tween 20 (TBS-T) and 5% non-fat dry milk for 1 h at 4°C and then in TBS-T with 0.1% (v/v) rabbit antiserum for 16 h at room temperature. Antiserums were obtained by immunization of rabbits with either P5CS or ProDH recombinant proteins (Thiery et al., 2004). Blots were washed with TBS-T. Detection was performed with an ECL assay using horseradish peroxydase-conjugated secondary antibodies (GE Healthcare). Equal protein loading and integrity of protein samples were verified by Ponceau S red staining of the blot membrane.

### Metabolite profiling using GC-MS and metabolomics data processing

Three independent samples of 12-days-old seedlings from each genotype treated during 24 h in different conditions were collected, and the equivalent of 50 mg of powder of each samples were used to perform the extraction and further metabolomics analysis. Extraction, derivatization, analysis, and data processing were performed according to Fiehn (2006). Metabolites were analyzed by GC-MS 3 h after derivatization. One microliter of the derivatized samples was injected in splitless mode on an Agilent 7890A gas chromatograph coupled to an Agilent 5975C mass spectrometer. The column was an Rtx-5SilMS from Restek (30 m with 10-m Integra-Guard column). The liner (Restek 20994) was changed before each series of analyses, and 10 cm of column was removed. The oven temperature ramp was 70°C for 7 min then 10°C/min to 325°C for 4 min (run length 36.5 min). The helium constant flow was 1.5231 mL/min. Temperatures were as follows: injector, 250°C; transfer line, 290°C; source: 250°C; and quadripole, 150°C. Samples and blanks were randomized. Amino acid standards were injected at the beginning and end of the analysis to monitor the derivatization stability. An alkane mix (C10, C12, C15, C19, C22, C28, C32, and C36) was injected in the middle of the queue for external calibration. Five scans per second were acquired.

Metabolites were annotated, and their levels on a fresh weight basis were normalized with respect to the ribitol internal standard.

Raw Agilent data files were converted in NetCDF format and analyzed with AMDIS (http://chemdata.nist.gov/mass-spc/amdis/). A home retention index/mass spectra library built from the NIST, Golm, and Fiehn databases and standard compounds were used for metabolite identification. Peak areas were then determined using the Quanlynx software (Waters) after conversion of the NetCDF file to masslynx format. TMEV (http://www.tm4.org/mev.html) was used for all statistical analysis. Univariate analysis by permutation (One-Way and Two-Way ANOVA) was first used to select the significant metabolites. Multivariate analysis (hierarchical clustering and principal component analysis) was then performed on this subset.

## Results

### LY294002 affects proline accumulation only in response to salt treatment

**Figure 1:**
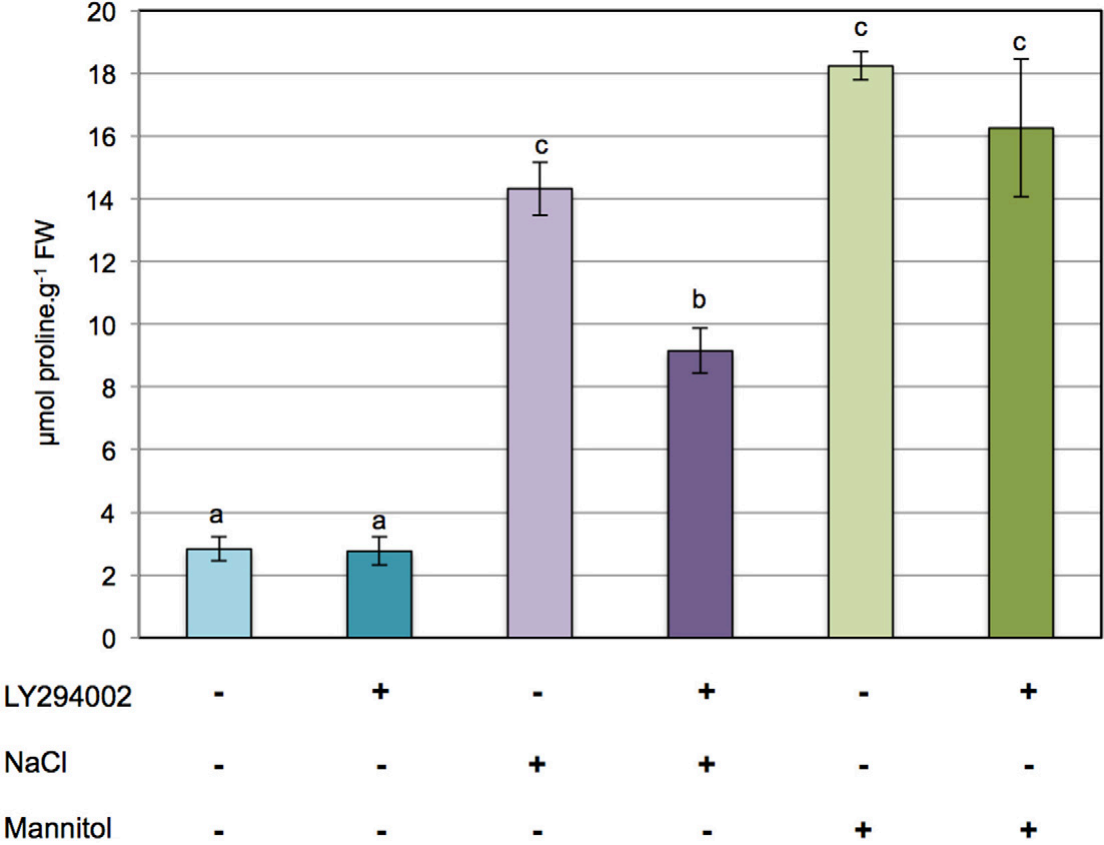
**LY294002 induces a decrease in proline levels only upon salt stress in Arabidopsis**. Twelve-days-old seedlings grown on 0.5× MS solid medium were transferred to 0.5× MS liquid medium for treatments. Plants were pre-incubated for 1 h with 100 μM LY294002 or with the same amount of DMSO as a control, and then stressed for 24 h with either 200 mM NaCl or 400 mM mannitol supplemented with 100 μM LY294002 (+) or with the same amount of DMSO as a control (−). The results shown are means ± SD of four independent samples. Letters indicate statistical differences in proline level depending on treatment conditions (Two-Way ANOVA test, *P* < 0.05). FW, fresh weight.

To investigate whether PI3K is involved in the regulation of proline metabolism in response to ionic and/or hyperosmotic constraints, the effect of LY294002 on proline accumulation was assessed in 12-days-old Arabidopsis seedlings subjected to either 200 mM NaCl or 400 mM mannitol for 24 h. As shown in Figure 1A, typically a 5- to 6-fold accumulation of proline is observed in Arabidopsis seedlings treated with either NaCl or mannitol in comparison to the control seedlings. Interestingly, while LY294002 had no effect on the proline levels in control seedlings or seedlings stressed with mannitol (Figure 1), 40% less proline accumulated in the LY294002-treated seedlings upon salt stress. When increasing concentrations of LY294002 were applied, proline levels decreased in a dose-dependent manner in plants stressed with NaCl, with a maximum effect observed for 100 μM LY294002 (Supplementary Figure 1). In contrast, no effect on the proline levels of control or mannitol-stressed seedlings where found, whatever concentration of LY294002 (Figure 1 and Supplementary Figure 1). These results show that LY294002 negatively regulates proline accumulation in response to salt stress but not to mannitol.

### LY294002 reduces the level of PI3P

In order to characterize the inhibitory effect of LY294002 on PI3K activity, *in vivo* PI3P levels were measured. For practical reasons, 6-days-old seedlings were used. These seedlings accumulated slightly less proline than the 12-days-old seedlings after NaCl treatment but the results were consistent, indicating that the younger seedlings perceived and responded well to salt stress (data not shown).

**Figure 2:**
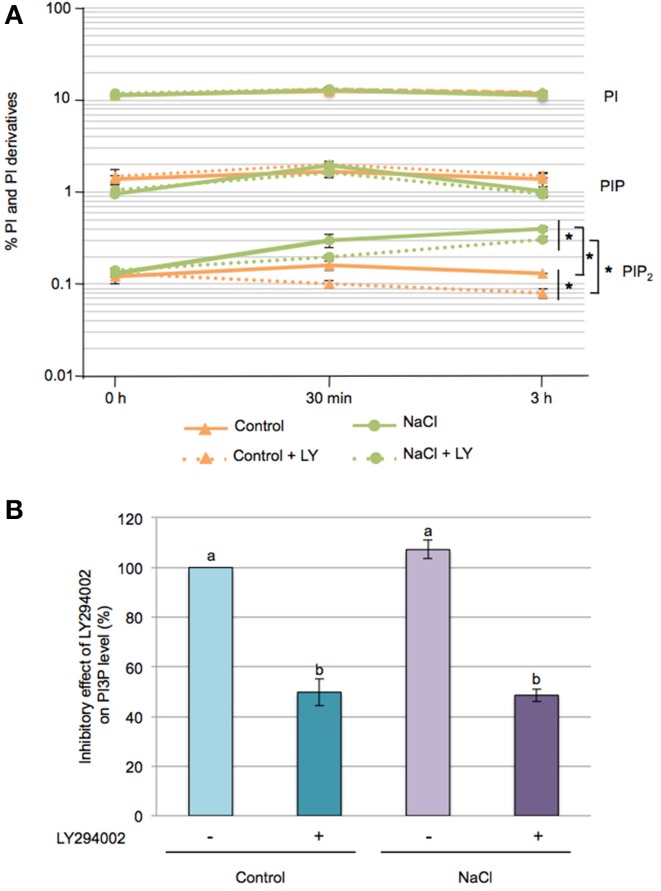
**Effect of LY294002 on the levels of Phosphatidylinositol and its derivatives upon salt stress**. Five-days-old seedlings were labeled overnight with ^32^P_i_, pre-incubated for 1 h with 100 μM LY294002 or with DMSO, then were stressed with 200 mM NaCl for 30 min or 3 h in the presence (+LY) or absence of LY294002. **(A)** Lipids were extracted, separated by TLC and quantified by PhosphoImaging. Data are expressed as percentages of PI, PIP and PIP_2_ of total ^32^P-phospholipids and are means ± SD of three independent samples containing three seedlings each. A semi-log scale was used for the representation. Significant differences between seedlings treated with or without 100 μM LY294002 are indicated by ^*^(Two-Way ANOVA test, *P* < 0.05). **(B)** After TLC separation, PIP spots were scraped-off, deacylated and the resulting GroPInsPs separated by HPLC and quantified by liquid-scintillation counting. The resulting graph represents the variation in PI3P levels at 30 min of treatment expressed as a percentage, with 100% corresponding to the control condition without LY294402. Results are means ± SD of three independents experiments. Letters indicate statistical differences in PI3P level depending on treatment conditions (Two-Way ANOVA test, *P* < 0.05).

Seedlings were ^32^P_i_-labeled overnight and the lipids extracted and separated by TLC (Supplementary Figure 2). Phosphoinositides (PI, PIP, and PIP_2_) were quantified using PhosphoImaging (Figure 2A). PI, a structural phospholipid of membranes, represented 11–12 % of the total ^32^P-labeled phospholipids, and its levels remained fairly constant throughout our experiments. PIP and PIP_2_ are minor lipid constituents and accounted for 1–2% and 0.1–0.15% of the total phospholipids, respectively. PIP_2_ progressively increased upon salt stress, reaching a 3-fold increase at 3 h compared to control seedlings, while no significant effects of salt stress were found for PIP levels. Interestingly, LY294002 treatment caused a slight but significant decrease in PIP_2_ under control conditions as well as in response to salt stress.

In plants, the PIP pool is composed of 3 isomers, PI3P, PI4P, and PI5P (Munnik and Vermeer, 2010). PI4P is the most predominant PIP species (~80–90%), with PI3P and PI5P each accounting for ~5–10% of the PIP pool (Meijer et al., 2001). On TLC, the PIP isomers cannot be separated but by removing their fatty acids and analysing the resulting glycerophosphoinositolphosphates (GroPInsPs) by HPLC, it is relatively easy to distinghuish and quantify the GroPIns3P from the GroPIns4P and GroPIns5P. The latter two are rather difficult to separate (Meijer et al., 2001).

So to determine the PI3P levels under our conditions, TLC-separated ^32^P-labeled PIP spots from 30 min treated seedlings were scraped off, deacylated and separated by anion-exchange HPLC. At control and salt conditions, PI3P was found to account for ~5% of the PIP pool. Addition of 100 μM LY294002, however, induced a 50% decrease of PI3P, whatever control or stress condition (Figure 2B). These results, and the inhibitory effect of LY294002 on proline accumulation in response to salt stress, are consistent with the involvment of PI3P as a lipid mediator on the regulation of proline metabolism.

### LY294002 impacts the expression of genes involved in proline metabolism

Proline accumulation is the consequence of a tight regulation of gene expression (Szabados and Savouré, 2010; Liang et al., 2013). We investigated transcript accumulation of two genes involved in proline metabolism, *P5CS1* and *ProDH1* that encode key enzymes regulating proline biosynthesis and catabolism, respectively. RT-qPCR analysis showed a 17-fold higher *AtP5CS1* transcript accumulation in seedlings upon 3 h salt stress than in control ones (Supplementary Figure 3), which lower to 3-fold at 24 h salt stress. In addition, salt stress induced a slight accumulation of *AtProDH1* transcript but only after 24 h (Supplementary Figure 3).

**Figure 3:**
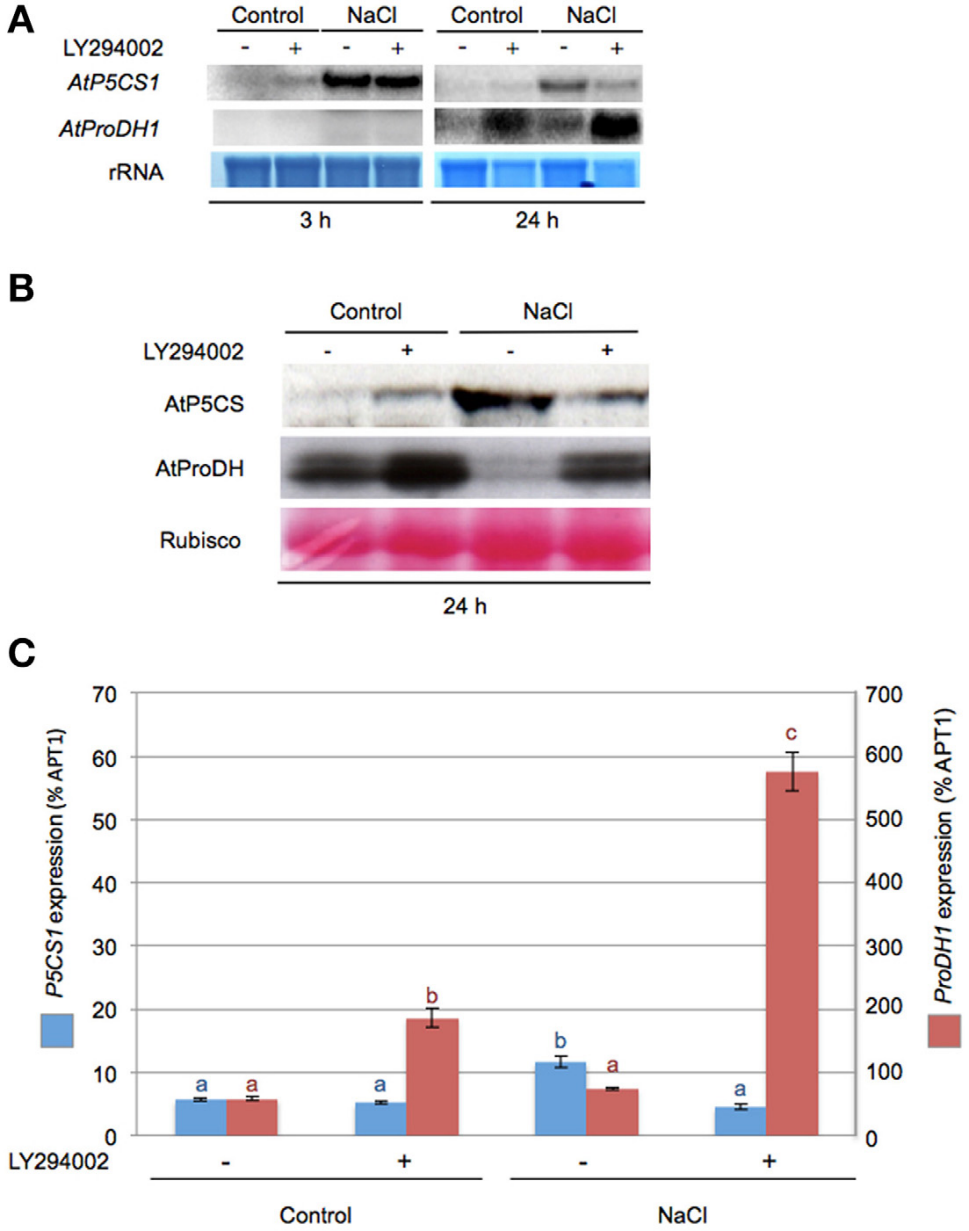
**LY294002 impacts *ProDH1* steady state transcript and proteins levels**. Northern- and western blot analysis were performed on total RNA or proteins extracted, respectively, from 12-days-old seedlings treated with either 0.5× MS alone (control) or supplemented with 200 mM NaCl (NaCl) during 3 h or 24 h with 100 μM LY294002 (+) or DMSO (−) as described in the legend of Figure 1. **(A)** Total RNA (10 μg) was loaded in each lane and northern blots were probed with DNA fragments specific for *AtP5CS1* and *AtProDH1*. Methylene blue staining of rRNAs is shown as a loading control. **(B)** Total proteins (20 μg) were separated on an 8% SDS-PAGE gel. Western blots were incubated with specific antibodies directed against AtP5CS and AtProDH proteins. Detection of the immunolabeled proteins was done by autoradiography using an ECL kit. Membranes were stained with Ponceau S Red as control for protein loading (Rubisco). **(C)** Relative expression levels of *P5CS1* and *ProDH1* genes measured by real-time quantitative PCR after 24 h treatment as described in **(A)**. Results expressed as percentage compared to *APT1* as a reference gene are means ± SD of 3 replicates. Letters indicate statistical differences in *P5CS1* (blue letter) or *ProDH1* (red letter) gene expression depending on treatment conditions (Two-Way ANOVA test, *P* < 0.05). The results presented in **(A,C)** were obtained from two independent experiments.

The role of PI3K on key genes and enzymes involved in proline metabolism was investigated using LY294002. Northern and western blot analysis revealed that LY294002 affected mRNA and protein accumulation in all tested conditions. After 3 h LY294002 treatment, a modest increase of *P5CS1* mRNA was observed in control condition while *ProDH1* transcripts were not detectable (Figure 3A). A dramatic effect of LY294002 on both *P5CS1* and *ProDH1* expression compared to non-treated seedlings was observed at 24 h salt stress. In salt stress seedlings treated with LY94002, *P5CS1* steady state transcript level was lower than in non-treated ones while *ProDH1* transcript level was higher (Figure 3A). In salt stress seedlings treated with LY294002, *P5CS1* transcript accumulation decreased by 60% (Figure 3C) compared to non-treated seedlings. RT-qPCR analysis confirmed the higher *ProDH1* transcript accumulation in presence of LY294002 whatever the growth conditions (Figure 3C). Using western blots, LY294002 triggered P5CS accumulation in control seedlings while P5CS level diminished in salt-treated plantlets. In contrast, LY294002 strongly enhanced ProDH accumulation in both control and salt-treated seedlings (Figure 3B).

The lower proline accumulation observed in response to salt stress with LY294002 is correlated with a down-regulation of *P5CS1* and up-regulation of *ProDH1* at both transcript and protein levels. As LY294002 reduced PI3P levels, our data suggest that PI3K is involved in the regulation of proline metabolism.

### *ProDH1* expression is induced in *pi3k* mutant

**Figure 4:**
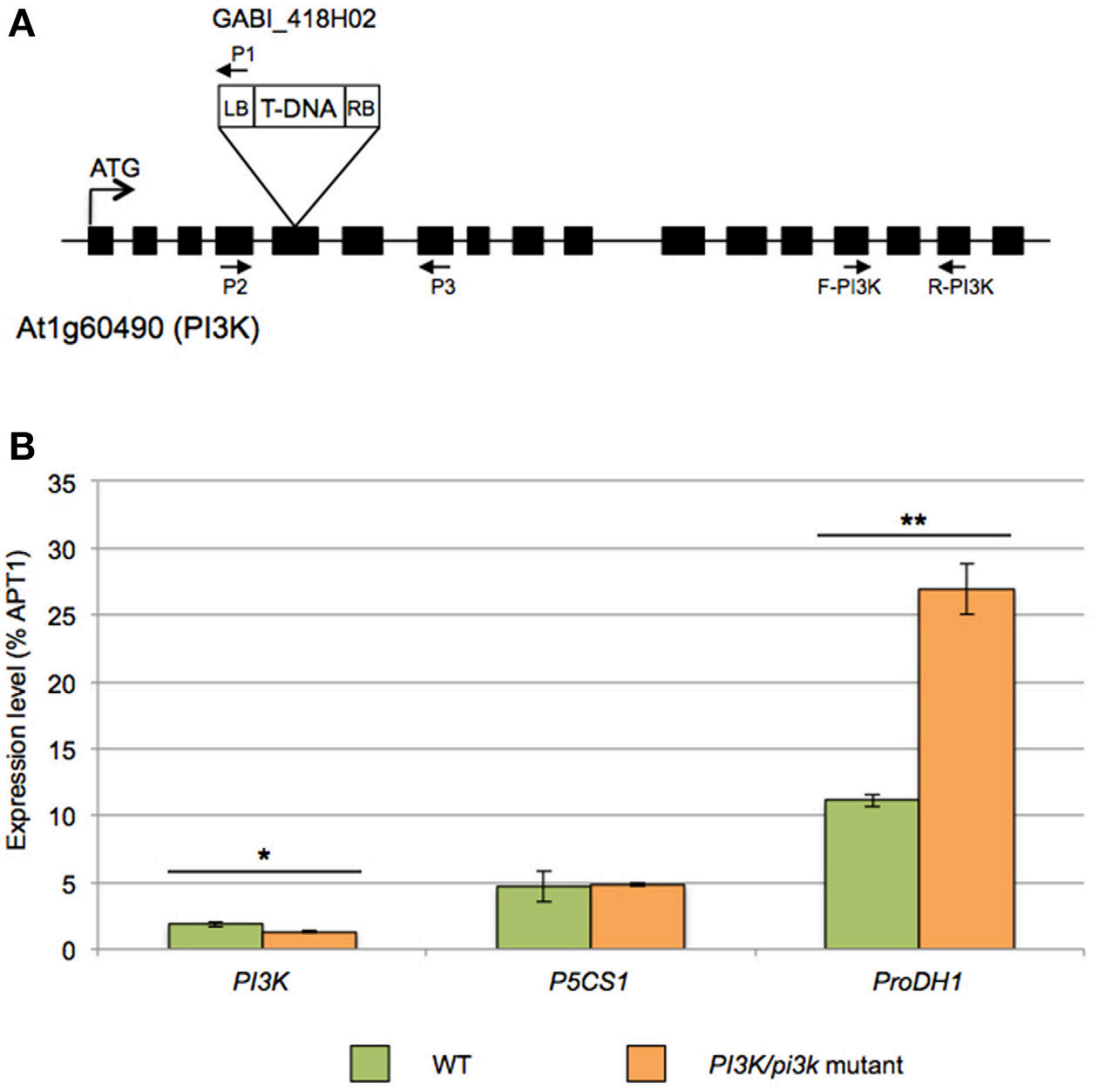
**Molecular characterization of the GABI_418H02 *pi3k* mutant. (A)** Schematic representation of the T-DNA insertion in *PI3K* gene (At1g60490). Boxes indicate exons. In GABI_418H02 mutant, T-DNA insertion is located in the fifth exon of the *PI3K* gene. Primer 1 (P1) corresponds to LB08409 primer specific to left border of the T-DNA and primers 2 (P2) and 3 (P3) are gene-specific primers used for the genotyping. **(B)** Expression analysis by RT-qPCR of *AtPI3K, AtP5CS1* and *AtProDH1* in WT and *PI3K/pi3k* hemizygous mutant. cDNA were obtained from 12-days-old WT and *PI3K/pi3k* seedlings. Results expressed as percentage compared to *APT1* as a reference gene are means ± SD (*n* = 3). Significant differences of gene expression between WT and hemizygous seedlings are indicated by ^*^(Student's *T*-Test, ^*^P < 0.05 and ^**^P < 0.01).

To further unravel the role of PI3K in the regulation of proline metabolism, we aimed for Arabidopsis *pi3k* KO mutants. Unfortunately, however, homozygous *pi3k* mutants are not viable (Lee et al., 2008b; Gao and Zhang, 2012). To partly resolve this issue, we selected sulfadiazine-resistant seedlings to get hemizygous *pi3k* mutant (Leshem et al., 2007; Lee et al., 2008a,b). Segregation analysis indicated a 1:1 ratio in sulfadiazine resistant and sensitive seedlings, respectively (data not shown), due to the male gametophytic defect (Lee et al., 2008b). Therefore *PI3K/pi3k* hemizygous mutants were selected and further analyzed (Figure 4A). PCR-based genotyping of GABI_418H02 *pi3k* mutants confirmed that no homozygous mutants were obtained from this selection (data not shown).

Expression analysis by RT-qPCR revealed a 25% decrease of steady-state *PI3K* transcript level in *pi3k* hemizygous mutants compared to WT (Figure 4B). In this mutant, *ProDH1* transcript level was almost 5-fold higher than in WT seedlings in normal growth condition. On the contrary, no difference in *P5CS1* transcript level was observed between WT and *pi3k* hemizygous mutant.

**Figure 5:**
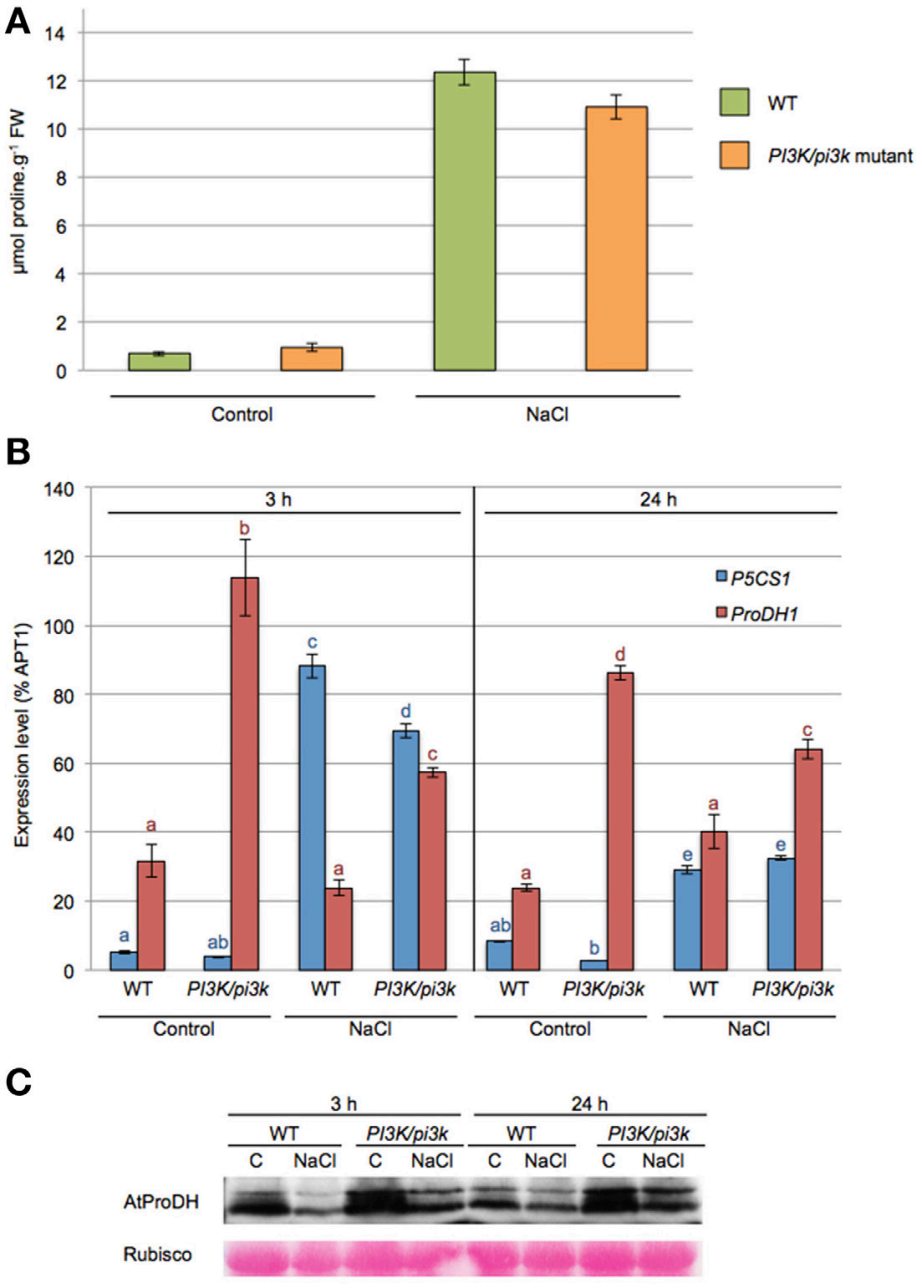
***ProDH1* transcript and protein levels are affected in *pi3k* hemizygous mutant**. Twelve-days-old WT and *pi3k* hemizygous mutant (*PI3K/pi3k*) seedlings were stressed with 200 mM NaCl (NaCl) or maintained in 0.5× MS (control, C) during 3 h or 24 h. **(A)** Proline levels were measured in 24 h-stressed seedlings. Results shown are means ± SD of 6 to 10 biological repetitions in two independent experiments. FW, Fresh Weight. **(B)** Expression levels of *P5CS1* and *ProDH1* genes by RT-qPCR on cDNA obtained from 3 h to 24 h- treated WT and *pi3k* hemizygous mutant seedlings. Results expressed as percentage compared to *APT1* as a reference gene are means ± SD (*n* = 3). Letters indicate statistical differences in *P5CS1* (blue letter) or *ProDH1* (red letter) gene expression in WT and *pi3k* hemizygous mutant depending on treatment conditions (Two-Way ANOVA test, *P* < 0.05). **(C)** Total proteins obtained from 3 h to 24 h-stressed WT and *pi3k* hemizygous mutant seedlings were separated by SDS-PAGE. Western blots were incubated with specific antibodies directed against AtProDH proteins. Detection of the immunolabelled proteins was done by autoradiography using an ECL^+^ kit. Membranes were stained with Ponceau Red as control for protein loading (Rubisco).

When WT and *pi3k* hemizygous mutant were subjected to 200 mM NaCl for 24 h, they showed a higher proline accumulation of 18-fold and 11.5-fold, respectively (Figure 5A). *pi3k* hemizygous mutant showed a lower proline accumulation in response to NaCl. However the proline accumulation was not significantly different from WT seedlings, probably due to the remaning *PI3K* wild-type allele.

*P5CS1* and *ProDH1* transcript levels were investigated in *pi3k* hemizygous seedlings in response to 3 and 24 h of salt stress (Figure 5B). *P5CS1* mRNA accumulation was similar in WT and *pi3k* hemizygous in response to salt stress. An equivalent increase of *P5CS1* transcripts was observed at 3 h of stress and a decrease at 24 h of stress. Higher *ProDH1* transcript levels than WT were observed in *pi3k* mutant in all tested conditions whether the seedlings were subjected to salt stress or not (Figure 5B). In *pi3k* hemizygous mutant, ProDH protein levels were stronger than in WT at both 3 and 24 h salt stress (Figure 5C). These results indicate that *pi3k* hemizygous seedlings are able to respond to salt stress, triggering *P5CS1* gene expression and proline accumulation, although the transcript level of *ProDH1* gene is increased. Alltogether, our data indicate that the higher level of *ProDH1* transcripts is correlated with higher ProDH amount in *pi3k* mutant. These data are consistent with those obtained with LY294002 treatment (Figure 3), where LY294002 induced higher ProDH1 transcripts and proteins. These results further strengthen the participation of a PI3K-mediated pathway regulating proline catabolism.

### LY294002 affects seedling metabolome

**Figure 6:**
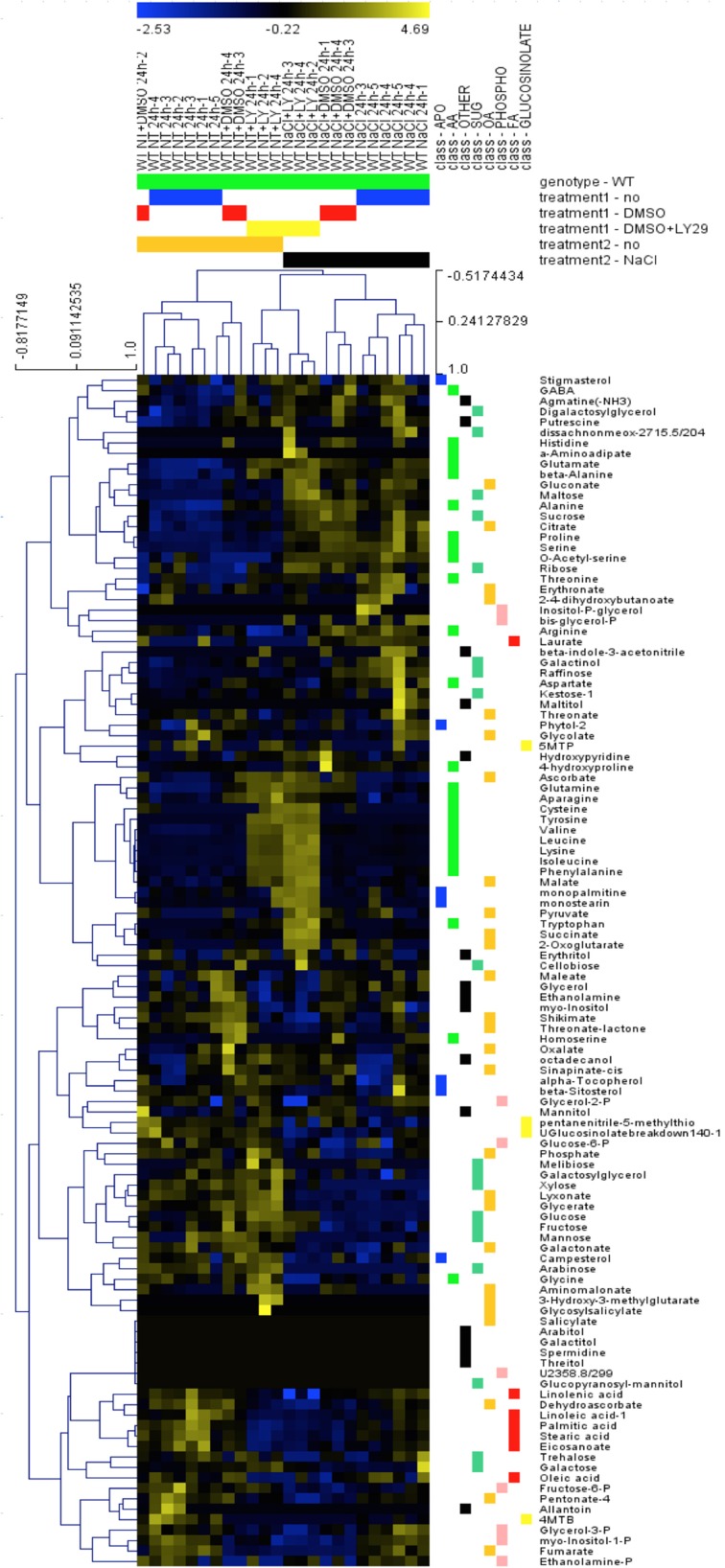
**Hierarchic clustering of metabolite changes in WT seedlings subjected to NaCl and LY29002**. Metabolite relative content was investigated in 12-days-old Arabidopsis wild-type seedlings treated during 24 h as explained in Materials and Methods and analyzed using hierarchic clustering tool (MEV4.0). The color scale shows the concentration ratios in log2.

**Figure 7:**
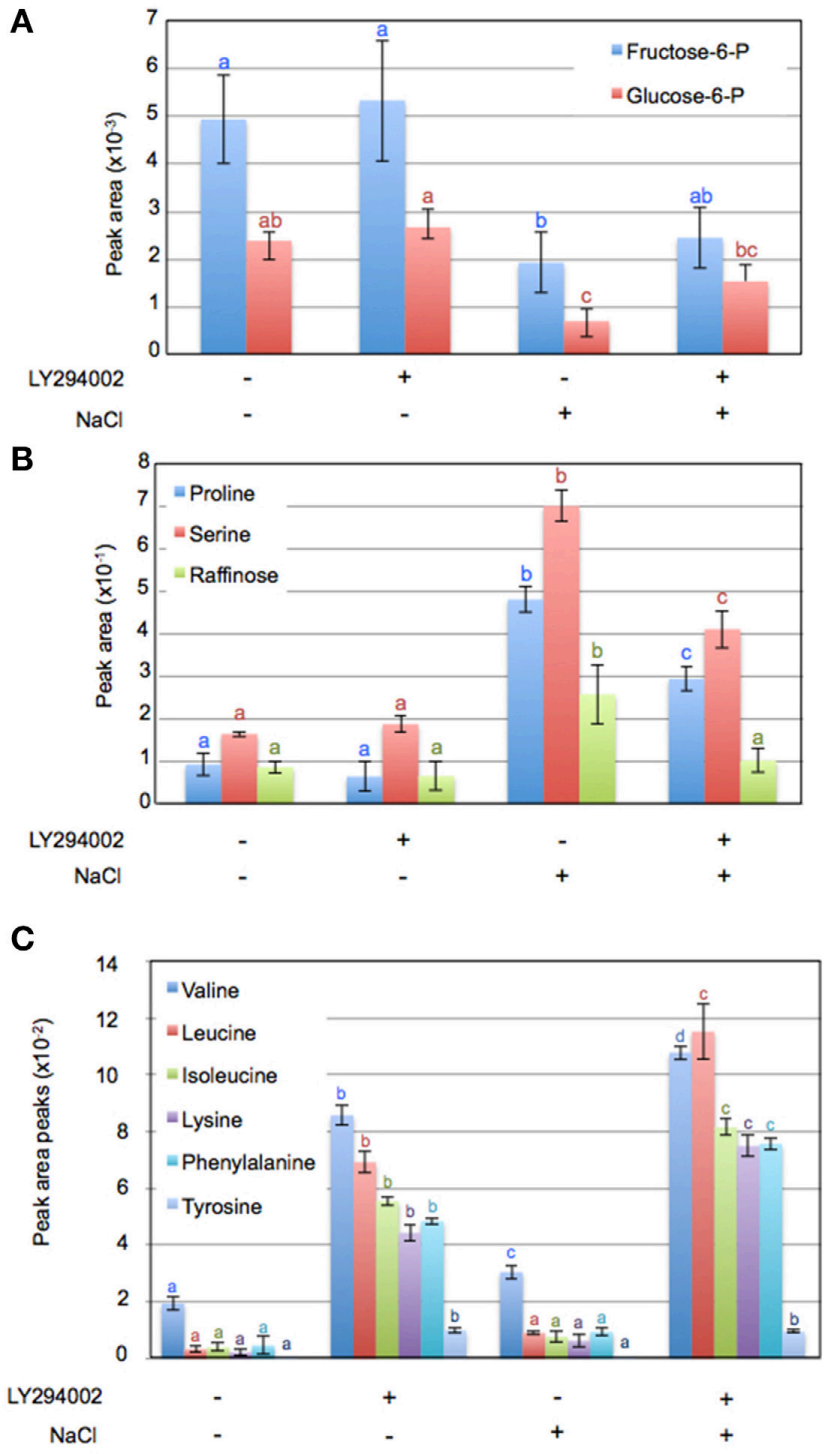
**Variation of metabolite content in Arabidopsis seedlings in response to salt stress with or without LY294002**. Metabolite contents of 12-days-old Arabidopsis wild-type seedlings treated during 24 h as explained in Materials and Methods. Values in **(A–C)** are computed from normalized areas of specific peaks after GC-MS experiments (see Materials and Methods for details) and are the means ± SD of three independent repetitions. Letters in **(A–C)** indicate statistical differences in metabolite levels depending on treatment conditions (Two-Way ANOVA test, *P* < 0.05).

To investigate other changes induced by LY294002, we compared the metabolite profiles of 12-days old WT seedlings treated with LY294002 or DMSO upon control and salt stress conditions. Hierarchic clustering analysis indicated that DMSO did not have any significant effect on the relative metabolites contents, indicating that the difference in the metabolite patterns obtained with LY294002 is an effect of the inhibitor (Figure 6). Treatment for 24 h of salt stress significantly modified the amounts of several metabolites. Relative amounts of sucrose, ribose and maltose as well as proline, serine and raffinose increased in response to salt stress (Figures 6, 7B). On the contrary, the amounts of some other sugars, like galactose, mannose, trehalose and xylose (Figure 6) and glucose-6-phosphate (Glucose-6-P) and fructose-6-phosphate (Fructose-6-P) decreased in response to salt stress (Figure 7A). LY294002 reduced the level of proline content almost by half (Figure 7B), in accordance with our previous results (Figure 1 and Supplementary Figure 1). Interestingly, two other compatible osmolytes, serine and raffinose, exhibited an accumulation pattern similar to that of proline, i.e., higher accumulation in response to salt stress and lower when LY294002 is added (Figure 7B). Surprisingly, LY294002 addition had a strong impact on some amino-acid levels whatever the treatment. The most dramatic effect being for tyrosine with an almost 100-fold increase in the presence of LY294002, in either control condition or NaCl stress (Figure 7C). Similarly, a 10–20-fold increase was observed for lysine, leucine, isoleucine and phenylalanine and a 4-fold increase for valine in response to LY294002.

Thus, the strong increase of those aliphatic and aromatic amino acids in presence of LY294002 suggests that PI3K, and/or its product PI3P, negatively regulates their metabolism through inhibition of their synthesis and/or promotion of their catabolism.

## Discussion

In this paper, we investigated the effect of the PI3K inhibitor, LY294002, on the response of Arabidopsis seedlings to salt stress. LY294002 was found to reduce PI3P levels by 50% and to dramatically decrease the accumulation of proline upon salt stress. The latter was a consequence of lower transcript- and protein levels for P5CS1 and higher transcript and protein levels for ProDH1. In the *pi3k* hemizygous mutant line, as also observed for WT seedlings treated with LY294002, an up-regulation of *ProDH1* expression was found, suggesting that PI3K and its product PI3P are involved in a pathway repressing *ProDH1* expression. Metabolomic profiling of Arabidopsis seedlings in response to salt stress showed that LY294002 reduced the amount of raffinose, another compatible osmolyte, and strongly increased the amount of free aliphatic and aromatic amino acids.

### NaCl stress modified phosphoinositide composition

PI is not only a structural phospholipid of membranes, but also a precursor of several signaling phosphoinositides that are produced by distinct kinases and phosphatases, which add and remove phosphates at different positions of the inositol ring (Mueller-Roeber and Pical, 2002; Xue et al., 2009; Munnik and Vermeer, 2010; Munnik and Nielsen, 2011). Characterization of the phospholipid composition of overnight ^32^P-labeled Arabidopsis seedlings showed that salt stress mostly affected the PIP_2_ pool. This latter is predominantly composed of the PI(4,5)P_2_ isomer (Munnik, 2013). Some plant systems, in particular *Chlamydomonas*, have reported on increased PI(3,5)P_2_ levels (Meijer et al., 1999), but we did not observe this here for Arabidopsis seedlings. Increased PIP_2_ levels in response to salt and/or osmotic stress have been reported for several plant systems (Pical et al., 1999; DeWald et al., 2001; Zonia and Munnik, 2004; Darwish et al., 2009; Munnik and Zarza, 2013). Part of this PI(4,5)P_2_ response occurs at the plasma membrane (Van Leeuwen et al., 2007; König et al., 2008), where it is considered to be an important signaling molecule (Munnik and Nielsen, 2011), potentially through initiation of vesicle budding *via* its interaction with clathrin-adapter proteins. The subsequent formation of clathrin-coated vesicles during salt stress could be a mechanism for the cell to modify the plasma membrane according to water/ion movement (König et al., 2008). E.g., PI(4,5)P_2_ has been suggested to modulate stomatal aperture in response to water stress by regulating K^+^-efflux channel (Lee and Lee, 2008). Alternatively, PI(4,5)P_2_ can be hydrolysed by PLC to form inositol trisphosphate (IP_3_) and DAG, which can both be rapidly metabolized into other signaling molecules, e.g., IP_6_ and phosphatidic acid (Munnik and Vermeer, 2010). PLC has been implicated in salt stress signaling (Drøbak and Watkins, 2000; Tasma et al., 2008; Xue et al., 2009; Munnik and Vermeer, 2010). In addition, Parre et al. (2007) have demonstrated that proline biosynthesis in response to salt stress is regulated by a Ca^2+^-signature depending on PLC activity.

LY294002 addition had a small effect on the PIP_2_ pool. Theoretically, as an ATP analog, LY294002 could inhibit other enzymes (Davies et al., 2000; Walker et al., 2000; Gharbi et al., 2007), but to our knowlegde there is no report regarding such an effect on PIP 5-kinases. Another explanation could be that vesicles containing the LY294002-sensitive PI3P pool require the downstream synthesis of PIP_2_, for example, to fuse with or pinch-off from membranes.

Minor changes in PIP levels were measured in *Arabidopsis* seedlings upon salt stress. This pool is a mixture of 3 isomers, i.e., PI3P, PI4P, and PI5P. The latter isomer is thought to result from PI(3,5)P_2_ dephosphorylation and is, like its precursor, present at very low concentrations (Meijer et al., 2001; Munnik, 2013). Since the PIP isomers cannot be separated by TLC, we removed the fatty acids by chemical deacylation and analyzed the resulting GroPInsP isomers by anion-exchange chromatography. As such, we found that only 5% of the PIP pool accounted for PI3P. The majority of the pool was composed of PI4P pool (>90%). PI5P levels were not determined as they were extremely difficult to separate from the 4-isomer (Meijer et al., 2001). LY294002 caused the PI3P pool to be reduced by 50%. No variation was observed in response to salt stress. Earlier, Meijer et al. (2001) reported an increase in PI3P after 5 min of 300 mM NaCl but this was in *Chlamydomonas*, which seems to exhibit a big difference in PI3P- and PI(3,5)P_2_ metabolism compared to higher plants. To our knowledge, there is no other data available on the effect of salt on PI3P levels. Nevertheless, several studies have indicated a role for PI3P in the plant's response to salt or water stress on the basis of PI3K inhibitors. As such, PI3P has been implicated in the regulation of stomatal closure in response to ABA (Jung et al., 2002). Leshem et al. (2007) have demonstrated that PI3P triggers the endocytosis of NADPH oxidase located at the plasma membrane under salt stress and this was implicated in the formation of ROS, which are important signaling molecules for plants to cope with salt stress (Leshem et al., 2006; Ben Rejeb et al., 2014). Phosphoinositides recruit proteins through specific phosphoinositide-binding domains to particular membranes (Van Leeuwen et al., 2004; Banerjee et al., 2010; Munnik and Nielsen, 2011). As such, the interaction between PI3P and the immunophilin ROF1 could participate in the plant's response to salt stress too (Karali et al., 2012). In addition, PI3P can participate in the regulation of vesicular trafficking and vacuole formation by recruiting proteins such as VTI11 and EPSIN that are involved in membrane fusion (Lee and Lee, 2008; Lee et al., 2010; Zheng et al., 2014a,b).

### PI3P is involved in the repression of proline catabolism

Proline accumulation is a well-known plant response to salt, and more generally, to water stress (Szabados and Savouré, 2010; Gupta and Huang, 2014). We found that LY294002 reduced the proline accumulation in response to salt stress. This could be explained by repression of proline biosynthesis and induction of proline catabolism genes both at the RNA and protein levels. The strong induction of *ProDH1* expression and the accumulation of the corresponding protein were correlated with the inhibitory effect of LY294002 on PI3P levels in both control and stress conditions, suggesting a role of PI3P on the inhibition of proline catabolism whatever the conditions. This hypothesis was confirmed by studies on *pi3k* mutant. *ProDH1* expression was higher in *pi3k* mutant than in the WT upon salt stress but also in normal growth condition. On the contrary, *P5CS1* expression while being diminished by LY294002 was not affected in *pi3k* mutant, suggesting that LY294002 may also act on other protein kinase activity involved in signaling pathway regulating *P5CS1* expression. We previously found evidence that *P5CS1* expression in response to salt stress fell under the regulation of PLC activity (Parre et al., 2007). Here, seedlings treated with LY294002 had a lower PIP_2_ response with salt. Maybe the lower availability of PIP_2_ as PLC substrate contributes to the reduced *P5CS1* expression. The comparison between seedlings treated with LY and *pi3k* mutant showed differences in *P5CS* expression in contrast to *ProDH* expression. This may be due to the fact that LY294002 could have additional effects like inhibiting other kinases. Another possibility is that adaptation to long-term decrease in PI3K activity could occur in the hemizygous *pi3k* mutant whereas the effect of LY294002 is more sudden and could change some of the plant stress responses.

Lee et al. (2008b) indicated that the *pi3k* mutant is strongly impaired in its pollen development, leading to the inability to obtain homozygous mutant. Our segregation analysis also showed a gametophytic defect in *pi3k* mutant, which explained why only *pi3k* hemizygous mutant could be obtained. *pi3k* mutant showed a reduced expression of the complete gene, in agreement with Leshem et al. (2007) and to the lethal phenotype of the reported antisense transformation (Welters et al., 1994). Consequently, genetic and biochemical analyses of PI3K are very difficult to assess. The development of promoter inducible lines may be useful to further investigate the role of PI3K in plant stress adaptation.

*ProDH1* expression is under the control of bZIP10 and bZIP53 transcription factors (Satoh et al., 2004; Weltmeier et al., 2006; Dietrich et al., 2011; Veerabagu et al., 2014). Their expression and activity is regulated by various abiotic constraints, and also by the nutrient status of the plant (Weltmeier et al., 2009). In mammals, nutrient deficiency induces *ProDH* gene expression as a consequence of mTOR complex inactivation (Liao et al., 2008). VPS34 has been shown to participate in the regulation of mTOR upon nutrient stress (Backer, 2008). TOR is a protein kinase that possesses a catalytic domain with strong homology to the kinase domain of PI3K, and has also been shown to be sensitive to LY294002 (Brunn et al., 1996). In Arabidopsis, it is therefore possible that LY294002 inhibits TOR kinase too and as a result ProDH. Nevertheless, the fact that the ProDH1 increase is also observed in the *pi3k* hemizygous mutant background supports a direct involvement of the PI3K pathway in repressing *ProDH1*. TOR could even be a downstream component of PI3K and as such participate in the regulation of *ProDH* expression. This has already been observed in other eukaryotes (Liao et al., 2008).

### Besides proline accumulation, LY294002 affects plant metabolism

Salt stress affects several metabolites, among them sugars such as hexose-phosphates, disaccharides and raffinose family oligosaccharides (RFO). Sucrose, maltose and raffinose accumulated in response to salt stress, whereas Glu-6-P and Fru-6-P decreased. Variations of these sugar contents are well-conserved responses among various plant species upon salt stress (Kempa et al., 2008; Sanchez et al., 2008). These sugars are derived from photosynthesis activity. They have a key role in osmoprotection, in ROS scavenging and as a source of carbon storage (Keunen et al., 2013; Gupta and Huang, 2014). Sugar could also be considered as signaling molecules regulating gene expression and triggering specific responses to abiotic stress (Keunen et al., 2013). Raffinose is the only sugar whose accumulation was reduced by 50% in salt-stressed Arabidopsis seedlings treated with LY294002. Similar to proline, raffinose is also considered as a compatible solute for plant cells (Valluru and Van den Ende, 2011; Van den Ende, 2013). In order to determine whether the biosynthesis of raffinose upon salt stress is dependent of the same signaling pathway as proline, it will be of interest to investigate the regulation of raffinose metabolism in *pi3k* mutant background.

The accumulation of several amino acids other than proline was observed upon salt stress. Aliphatic (Val, Leu, Ileu) and aromatic (Phe, Tyr) amino acids increased in response to salt stress (our study; Kempa et al., 2008; Sanchez et al., 2008), but also after an extended period of darkness (Usadel et al., 2008). These authors suggested that these might be due to induction of protein catabolism and/or remobilization of nitrogen sources. Surprisingly, a dramatic increase of these amino acids was observed in seedlings treated with LY294002 grown in normal conditions. Further experiments will be required to determine the role of PI3K signaling in the regulation of amino acid metabolism.

In conclusion, our results strongly indicate that a signaling pathway involving PI3K and its product PI3P is involved in the repression of proline catabolism upon salt stress. Identifying specific PI3P targets will allow to decipher whether TOR is an intermediate signaling component in the regulation of ProDH in plants.

### Conflict of interest statement

The authors declare that the research was conducted in the absence of any commercial or financial relationships that could be construed as a potential conflict of interest.
