# Supplementary material for: Involvement of Phosphatidylinositol 3-kinase in the regulation of proline catabolism in Arabidopsis thaliana
Source: Front Plant Sci. 2015 Jan 12;5:772. doi: 10.3389/fpls.2014.00772 (PMC4290513; doi:10.3389/fpls.2014.00772)
Supplement: Supplementary file 1 [file Presentation1.PDF]

Supplementary Table 1 : Primer sequence information

Primer name	primer sequence 5'--> 3'
F-P5CS1	GAGCTAGATCGTTCACGTGCTTT
R-P5CS1	ACAACTGCTGTCCCAACCTTAAC
F-ProDH1	CGGGATTGCGTAAAGAGAGA
R-ProDH1	GCCACAGGAAATGTTCTTACC
F-APT1	GAGACATTTTGCGTGGGATT
R-APT1	CGGGGATTTTAAGTGGAACA
F-PI3K	TGGCATTGGAGACAGACAC
R-PI3K	TGTAGGCTTCGCAACAG
P1	ATATTGACCATCATACTCATTGC
P2	AAGCAGAAGCTAAGGCTCTGG
P3	ACTCCACTCCACACAACGAAG

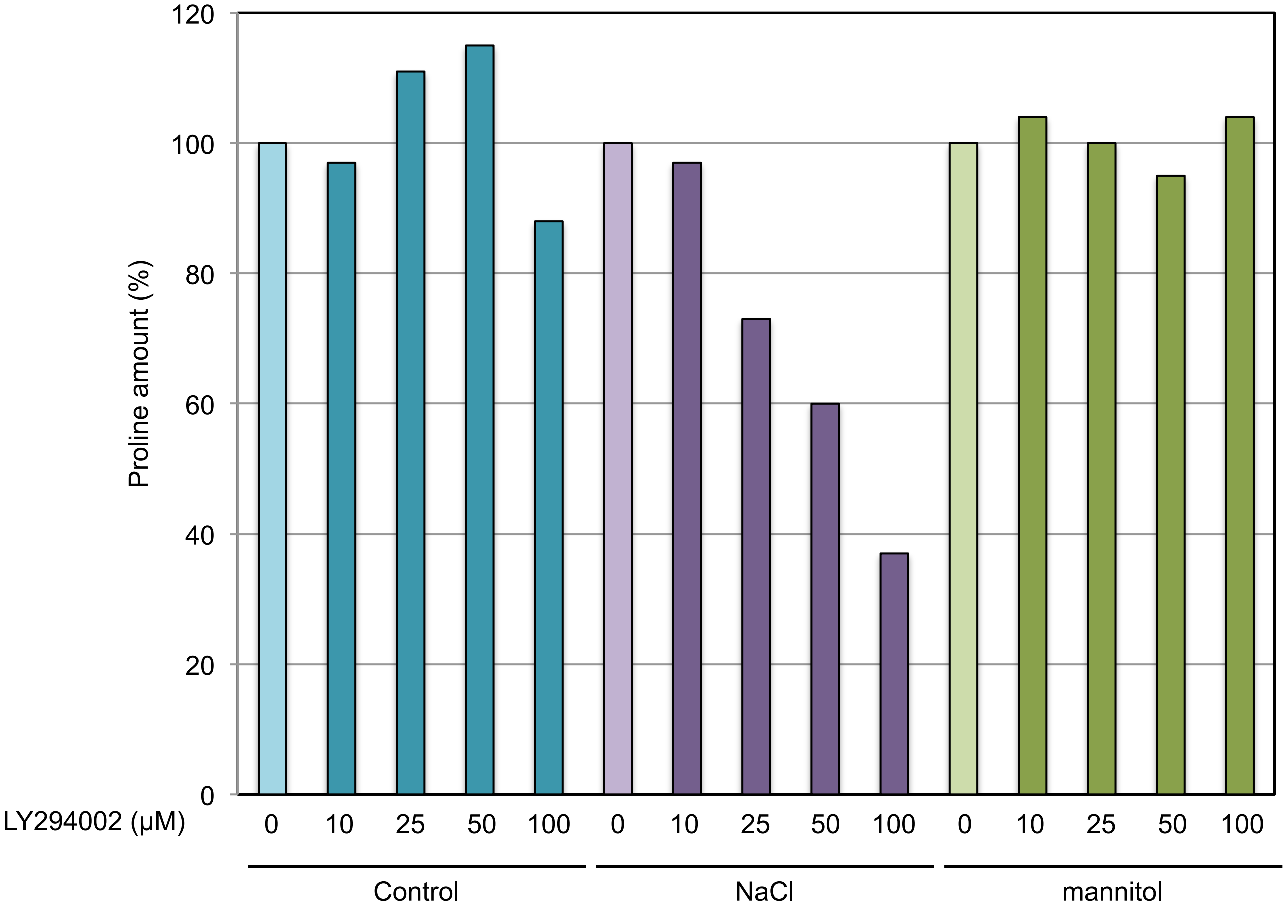

Supplementary Figure 1 : LY294002 induces a dose-response decrease on proline levels only upon salt treatment.

Plants were pre-incubated for 1 h with various concentrations of LY294002 (10, 25, 50 and 100 µM) or with the same amount of DMSO as a control (0), then treated for 24 h with either 200 mM NaCl or 400 mM mannitol and with either LY294002 or DMSO. The results are expressed as a percentage of proline levels compared to the condition without LY294002 corresponding to 100% for each treatment. The data have been obtained from three independent experiments.

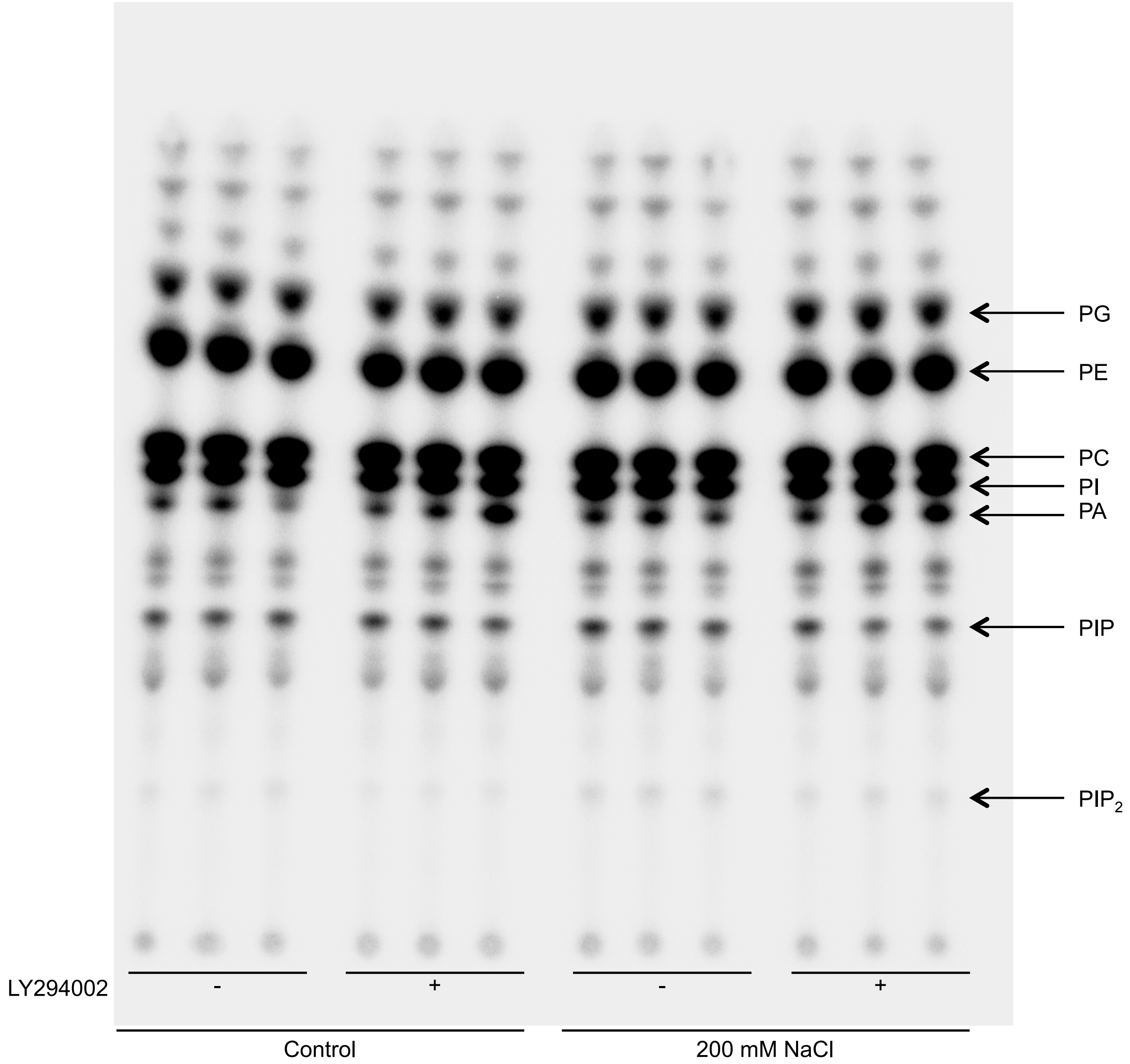

Supplementary Figure 2: Effect of 30 min of 200 mM NaCl with either or not 100 µM LY294002 on phospholipids.

Total ^{32}P-labelled lipids extracted from six-days-old Arabidopsis seedlings as described in Material and Methods were separated by one-dimensional TLC. PI : phosphatidylinositol, PIP : phosphatidylinositol monophosphate, PIP_2 : phosphatidylinositol diphosphate, PA : phosphatidic acid, PC : phosphatidylcholine, PE : phosphatidylethanolamine, PG : phosphatidylglycerol.

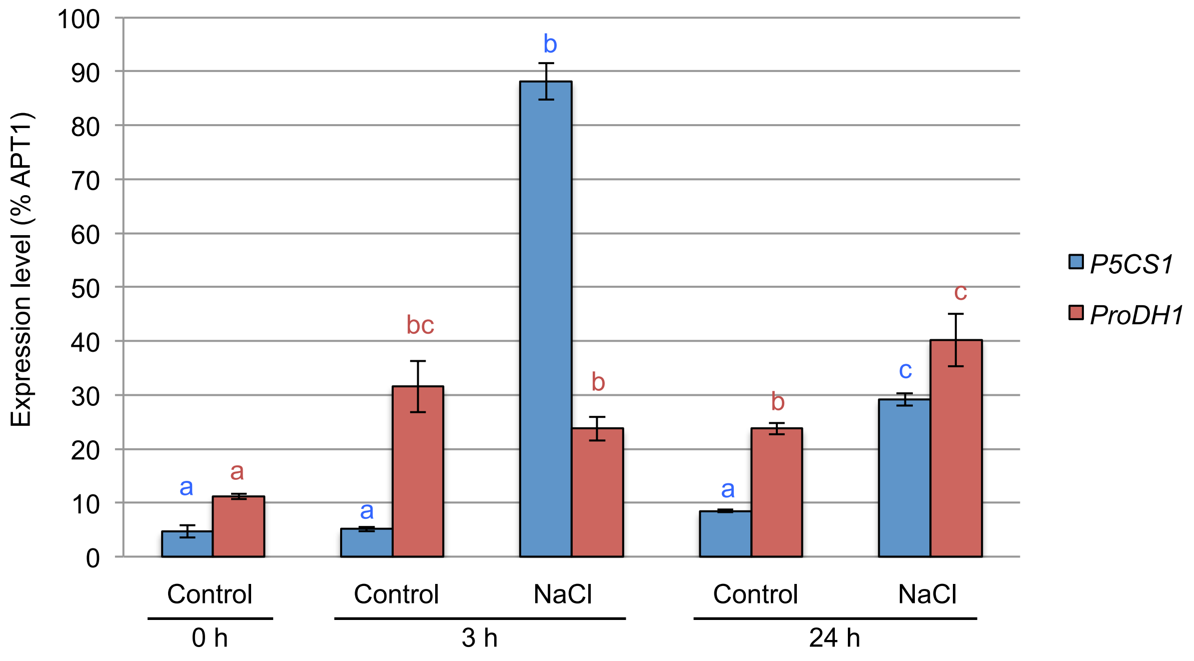

Supplementary Figure 3: *P5CS1* and *ProDH1* gene expression is regulated in response to 200 mM NaCl.

Expression of *P5CS1* and *ProDH1* genes is expressed as a percentage compared to *APT1* as a reference gene by RT-qPCR on cDNA obtained from 12-days-old WT seedlings before any treatment (0 h) or stressed with 200 mM NaCl during 3 h or 24 h in comparison to control conditions. Results are means ± SD (n=3). Letters indicate statistical differences in *P5CS1* (blue) or *ProDH1* (red letter) gene expression depending on culture conditions (two-way ANOVA test, $P<0.05$).
